# Supplementary material for: Occurrence and Genotypic Identification of Blastocystis spp., Enterocytozoon bieneusi, and Giardia duodenalis in Leizhou Black Goats in Zhanjiang City, Guangdong Province, China
Source: Animals (Basel). 2023 Aug 31;13(17):2777. doi: 10.3390/ani13172777 (PMC10486513; doi:10.3390/ani13172777)
Supplement: Supplementary file 1 [file animals-13-02777-s001.zip › Table S2. GenBank accession numbers of all SSU rRNA gene reference sequences of Blastocystis spp. used for phylogenetic analysis.pdf]

Table S2. GenBank accession numbers of all *SSU rRNA* gene sequences of *Blastocystis* spp. used for phylogenetic analysis (Figure 2), and associated information.

GenBank ID	Subtypes	Origin	Country	Genotype
KC148207	ST10	*Camelusdromedarius*	United Kingdom	ST10
MT042814	ST10	Sheep	Czech Republic	ST10
MK240481	ST10	Cattle	Malaysia	ST10
MK937750	ST10	Sheep	China	ST10
MZ265404	ST10	Goat	USA	ST10
AB070988	ST3	Human	Japan	ST3
MN526781	ST3	Sheep	United Kingdom	ST3
AB091235	ST3	Human	Japan	ST3
AY618266	ST1	Human	Thailand	ST1
AY618265	ST2	Human	Thailand	ST2
MN526815	ST14	Eurasianelk	United Kingdom	ST14
ON796560	ST14	Sheep	China	ST14
MW648930	ST14	Goat	Malaysia	ST14
ON796563	ST21	Goat	China	ST21
OM883875	ST21	Sheep	Denmark	ST21
OM883869	ST21	Muskox	Denmark	ST21
MW850524	ST5	Sheep	China	ST5
ON809458	ST5	Sheep	China	ST5
MK937752	ST5	Sheep	China	ST5
AY590115	not available	Sea snake	France	Outgroup
