# Supplementary material for: Occurrence and Genotypic Identification of Blastocystis spp., Enterocytozoon bieneusi, and Giardia duodenalis in Leizhou Black Goats in Zhanjiang City, Guangdong Province, China
Source: Animals (Basel). 2023 Aug 31;13(17):2777. doi: 10.3390/ani13172777 (PMC10486513; doi:10.3390/ani13172777)
Supplement: Supplementary file 1 [file animals-13-02777-s001.zip › Table S3. GenBank accession numbers of all ITS gene reference sequences of E. bieneusi used for phylogenetic analysis.pdf]

Table S3. GenBank accession numbers of all ITS gene sequences of *E. bieneusi* used for phylogenetic analysis (Figure 3), and associated information.

GenBank ID	Genotype	Origin	Country	Group
MH822618	CHG1	Goat	China	Group2
KU604931	CM21	Goldensnub-nosedmonkey	China	Group2
MT231509	ET-L2	Calf	Ethiopia	Group2
MK322762	BEB6	Tan sheep	China	Group2
MH937115	CGS1	Sheep	China	Group2
MH822618	CHG3	Goat	China	Group2
KP262366	CHG2	Goat	China	Group2
AF135836	I	Cattle	Germany	Group2
AF135837	J	Cattle	Germany	Group2
KR062124	WR8	*Apodemusagrarius*	Poland	Group9
KR062125	WR7	*Apodemusagrarius*	Poland	Group9
JF681175	KB-1	Baboon	Kenya	Group8
HM992511	CHN4	Human	China	Group1
AF242478	type IV	Human	France	Group1
AF101200	D	Human	Germany	Group1
MH817462	CTS3	Tibetan sheep	China	Group1
OM101102	PigSpEb2	Cattle	China	Group1
MT193871	EbpC	Dairy calves	China	Group1
JN997480	Nig4	Human	Nigeria	Group6
AY237212	WL4	Muskrat	USA	Group3
AY237214	WL6	Muskrat	USA	Group3
KT267287	CHK2	Gray kangaroo	China	Group10
KM591952	XH7	*Saimirisciureus*	China	Group7
KJ728811	CM18	*Lemurcatta*	China	Group7
JQ863274	WW6	Wastewater	USA	Group4
AY237209	WL1	Raccoon	USA	Group4
JF681180	KB-6	Baboon	Kenya	Group5
DQ683757	CAF4	Human	France	Group5
KJ668735	CD8	Dog	China	Group11
DQ885585	PtEb IX	Dog	Portugal	Outgroup
