# Supplementary material for: Occurrence and Genotypic Identification of Blastocystis spp., Enterocytozoon bieneusi, and Giardia duodenalis in Leizhou Black Goats in Zhanjiang City, Guangdong Province, China
Source: Animals (Basel). 2023 Aug 31;13(17):2777. doi: 10.3390/ani13172777 (PMC10486513; doi:10.3390/ani13172777)
Supplement: Supplementary file 1 [file animals-13-02777-s001.zip › Table S4. GenBank accession numbers of all bg gene reference sequences of G. duodenalis used for phylogenetic analysis.pdf]

**Table S4.** GenBank accession numbers of all *bg* gene sequences of *G. duodenalis* used for phylogenetic analysis (Figure 4), and associated information.

| GenBank ID | Genotype | Origin | Country | Assemblage |
|---|---|---|---|---|
| JX978265 | E | Goat | Brazil | Assemblage E |
| EU189361 | E | Goat | Spain | Assemblage E |
| MK452880 | E | Sheep | Greece | Assemblage E |
| MK573338 | E | Tibetan cattle | China | Assemblage E |
| MK573342 | E | Tibetan sheep | China | Assemblage E |
| KY769091 | E | Cattle | China | Assemblage E |
| MK610390 | E1 | Tan sheep | China | Assemblage E |
| KY432834 | E12 | Cattle | China | Assemblage E |
| AY647264 | F | Cat | Italy | Assemblage F |
| JX275388 | F | Cat | China | Assemblage F |
| KP687765 | AI | Human | Canada | Assemblage A |
| KM926506 | AI | Foal | Belgium | Assemblage A |
| FJ560591 | AI | Human | France | Assemblage A |
| EU769221 | G | *Rattusnorvegicus* | Sweden | Assemblage G |
| MF169196 | B | Horse | China | Assemblage B |
| MG736251 | B | Human | China | Assemblage B |
| KY979500 | D | Dog | China | Assemblage D |
| AY545647 | D | Dog | Italy | Assemblage D |
| MN044596 | C | Dog | China | Assemblage C |
| KY979502 | C | Dog | China | Assemblage C |
| LC437432 | C | Dog | Japan | Assemblage C |
| MT713338 | not available | Mouse | USA | Outgroup |
